# A descriptive retrospective study on HIV care cascade in a tertiary hospital in the Philippines

**DOI:** 10.1371/journal.pone.0281104

**Published:** 2023-01-31

**Authors:** Marisse Nepomuceno, Cybele Lara Abad, Edsel Maurice Salvaña

**Affiliations:** Division of Infectious Diseases, Department of Medicine, University of the Philippines–Philippine General Hospital, Manila, Philippines; Fairfield University Marion Peckham Egan School Of Nursing & Health Studies, UNITED STATES

## Abstract

**Introduction:**

The HIV care cascade is a model used to examine the engagement of people living with HIV (PLHIV) in medical care from the time of diagnosis to sustained viral suppression. This study describes the stages of the cascade from linkage to care, antiretroviral therapy (ART) initiation, retention in care, and virologic suppression- at the University of the Philippines—Philippine General Hospital (UP-PGH) STD/AIDS Guidance and Intervention Prevention (SAGIP) treatment hub in the context of existing cascades with similar demographics.

**Methods:**

We retrospectively reviewed the medical records of patients enrolled at the UP-PGH SAGIP treatment hub from June 2015 to December 2017. Baseline demographic and clinical data were collected, relevant to each stage of the cascade. Descriptive statistics using Microsoft Excel version 16.0 was used to characterize data and cumulative and conditional proportions were reported.

**Results:**

Of the 584 patients included in the cohort, majority were male (91.1%), with a median age of 29 years (range, 0.17 to 68 years). Male-to-male sex was the most common mode of transmission (325/584, 55.6%). Among all patients enrolled at the UP PGH SAGIP treatment hub, 99.5% were linked to care, 95.0% initiated ART, 78.8% were retained in care and maintained on ART, 47.9% were tested for HIV viral load, and 45.5% achieved viral suppression.

**Conclusion:**

A high proportion of patients enrolled at the UP-PGH SAGIP treatment hub are linked to care and initiate ART, exceeding the set goal of 90%, which is higher than reported nationwide. However, there is a substantial decrease in the number of patients who are subsequently retained in care, tested for HIV viral load, and achieve viral suppression. Gaps in the cascade related to healthcare delivery need to be investigated further and addressed by future studies. We recommend implementation of a community-based, patient-centered approach in order to reach the goals of the HIV cascade, with particular focus on young, MSM-PLHIV.

## Introduction

The HIV care cascade is a model examining engagement in medical care and the stages of care that people living with HIV/AIDS (PLHIV) experience from the time of their HIV diagnosis to sustained viral suppression. It is a useful tool to understand and examine the gaps and progress in HIV care [1–3]. In 2014, the UNAIDS aimed for the “90-90-90” target—90% of PLHIV should be aware of their status, 90% of those diagnosed should be on antiretroviral treatment (ART), and 90% of those on treatment should be virally suppressed by the year 2020 [4].

The UNAIDS Asia-Pacific region contributes the second highest number of PLHIV in the world. In this region, the epidemic is concentrated among key populations including men who have sex with men (MSM), transgender women who have sex with men, female sex workers, and people who inject drugs (PWID). Although there is an overall decline in the number of new infections around the Asia-Pacific by 31% from 2001–2014 [5], this was counterbalanced by the rapid rise of new infections in other countries including the Philippines [6, 7]. In fact, the Philippines has the fastest growing HIV epidemic in the Asia-Pacific region [8] and it is estimated that MSM make up 82% of all HIV cases [8], and contribute 90% of new cases [8]. The latest data from the Department of Health (DOH) Epidemiology Bureau HIV/AIDS and ART Registry in the Philippines (HARP) reported 1,472 new HIV seropositive individuals in June 2022 alone. This brings the total reported cases to 101,768 since January 1984, with 15% (14,796) having advanced clinical manifestation at the time of diagnosis [9]. In 2022, 42 cases of HIV are diagnosed per day, compared to 9 cases/day in 2012 [10]. This rapid rise of HIV in the Philippines is driven by multiple factors, including lack of access to care and barrier contraception, the war on drugs which has driven intravenous drug users to resort to needle sharing and other poor practices, and the stigma and discrimination surrounding HIV/AIDS [11].

As a response to the alarming HIV epidemic in the country, the Philippine Health Sector Plan adopted the UNAIDS “90-90-90” target. Although numbers are likely underestimated, data from the epidemiology bureau of the Philippines reported that the HIV care cascade from January 1984 to December 2020 showed that only 78,291 of 115,000 (68%) of the estimated PLHIV in the Philippines are aware of their status. Among those diagnosed, only 47,977 (61%) are alive and on ART. And although 94% of those with viral load determination are virally suppressed, only 8,155 (17%) of diagnosed PLHIV on ART by the end of 2020 were tested for HIV viral load [8]. To meet these challenges, the 6^th^ AIDS Medium Term Plan (AMTP) and the 2017–2020 Health Sector Plan provided a four-pronged approach which include 1) increasing knowledge and 2) preventing new infections among young key populations; 3) testing and treating 90% of PLHIV; and 4) eliminating mother-to-child transmission [8]. The 7^th^ AMTP aims to continue this approach but also emphasized the need to 1) protect the rights of PLHIV, key populations, and vulnerable communities; 2) strengthen system, governance, and leadership accountabilities and; 3) sustain a harmonized, fully resourced, and crisis-resilient HIV response [12].

The published data on HIV care cascades primarily reflect experiences among heterosexual populations, which may not be reflective of, or relevant in the Philippines where the overwhelming majority of HIV patients are MSM. A more recent study by Eustaquio *et al*. looked at the care cascade outcomes among MSM in a key population-led, community-based, HIV test-and-treat center in Metro Manila. They found that they achieved higher rates than national outcomes as 78% initiated ART and 84% achieved VL suppression in their center [13].

In 2015, the University of the Philippines-Philippine General Hospital (UP-PGH) SAGIP clinic, an HIV treatment hub adopted the test-and-treat strategy which has been shown to significantly reduce HIV transmission and HIV-related morbidity and mortality [14, 15]. This study aimed to describe the HIV care cascade in a tertiary level, hospital-affiliated HIV clinic after adoption of the test-and-treat strategy. We aimed to determine whether the UNAIDS targets were achieved in our setting, and hypothesized based on anecdotal data and clinical experience, that virologic suppression and retention in care would be problematic. We also compared the cascade in the context of available cascades in the Philippines, or other cascades with similar populations (e.g., MSM).

## Methods

### Study design and rationale

We performed a descriptive, observational, retrospective study to describe the stages of the HIV cascade from linkage to care, antiretroviral therapy (ART) initiation, retention in care, and virologic suppression at the University of the Philippines—Philippine General Hospital (UP-PGH) SAGIP treatment hub.

### Study setting

The UP-PGH is a tertiary level, state-owned 1500 bed-capacity hospital located in the city of Manila. It is designated as the National University Hospital and is the national government referral center that caters to the underserved, including patients from rural areas all over the country. The SAGIP treatment hub, one of 13 HIV-treatment hubs in Metro Manila, is located within the UP-PGH and was established in 1997. A treatment hub is a hospital-based facility with an established HIV-care clinic providing prevention, treatment, care, and support services to PLHIV, including but not limited to HIV counselling and testing, clinical management, and patient monitoring. SAGIP caters to 1,667 PLHIV, approximately 2.1% of all diagnosed PLHIV in the country. Both walk-in and scheduled patients are received in the clinic, often after referral for treatment upon testing positive, either during an outpatient consult or in-patient admission in the hospital, or more commonly, upon referral from an outside clinic or hospital without its own HIV treatment hub. The hub is open on weekdays during office hours and also offers STD counseling, testing and treatment. It is run by four nurses, one pharmacist, two administrative assistants, two case managers, infectious diseases fellows-in-training, and infectious disease consultants. In 2015, the hub adopted the test and treat initiative advocated by the UNAIDS. Given the initiative, all UP-PGH clinicians were recommended to use point of care testing for at-risk hospitalized population (e.g., early diagnosis). Each reactive HIV test was then automatically sent out to the STD-AIDS Cooperative Central Laboratory (SACCL) for confirmatory testing, a process that usually takes 1 to 3 months. SAGIP providers were all expected to start ART as soon as possible (e.g., early treatment), closely follow up their patients (e.g., retention in care) and document virologic suppression within 1 year of starting ART.

### Data collection

Records of all pediatric and adult patients who tested positive for HIV and were enrolled at the UP-PGH SAGIP treatment hub from June 2015 to December 2017 were retrospectively reviewed by a study author (MJN). Patients for inclusion were identified from the unit’s written and electronic database as follows: 1) All adult PLHIV confirmed by HIV Western Blot; 2) patients who were initially screened and diagnosed in another institution but were enrolled at the UP-PGH SAGIP treatment hub for initiation or continuation of treatment, or 3) patients who were lost to follow-up, who were transferred to another treatment hub, or those who died within the study period were included in the study. Patients were excluded if, upon review of the records they were HIV-negative, or HIV-seropositive but not officially enrolled as part of the SAGIP treatment hub (e.g. under the care of another hub, but a parent of an HIV-exposed infant). Patient characteristics recorded were age, sex, mode of HIV transmission, WHO clinical stage, and baseline CD4 and viral load. Relevant dates (e.g. HIV diagnosis, first consult, initiation of ART, last follow-up or ART refill, HIV viral load determination, transfer to another treatment hub) and the study participants’ HIV viral load level were recorded to determine the outcomes at each stage of the HIV care cascade.

### Definition of terms [16–18]

*PLHIV*. A patient with reactive HIV ELISA confirmed by Western blot at the National Reference Laboratory-San Lazaro Hospital/STD AIDS Cooperative Central Laboratory (NRL-SLH/SACCL).

*Late HIV diagnosis*. A CD4 count greater than or equal to 200 cells/uL but less than or equal to 350 cells/uL.

*Advanced HIV disease at diagnosis*. A CD4 count less than 200 cells/uL or presence of an AIDS-defining illness.

HIV care cascade.

***Linkage to care*.** First consult with an HIV physician or any physician part of an HIV-treatment hub.

***Initiated ART*.** Patients initiated on ART regardless of combination therapy.

***Retained in care*.** Patients documented to follow-up within the last 6 months for an acute care consult, scheduled visit, or refill of antiretroviral medications OR follow-up on the last scheduled date assigned by the attending physician.

***Viral suppression*.** An HIV RNA level of <1,000 copies/mL at least one year after starting ART.

### Ethical considerations

All information obtained was kept securely in a cabinet with a designated lock and key, and access to files was only available to study authors. Patients were de-identified and known only to the primary investigator (MJN) who reviewed charts from March 1 to May 31, 2019 by collecting data of patients within the study period. Incomplete or missing data was verified using the SAGIP unit electronic database, if available, or recorded as such, and not replaced. Study data will be kept until May 31 in 2024. Our study received ethical approval from the Institutional Review Board of the University of the Philippines–Manila Research Ethics Board. We abided by the Declaration of Helsinki [19] in the conduct of the study and followed both the Philippine HIV/AIDS Policy Act RA 11166 and the Data Privacy Act. Informed consent was waived because all data were de-identified and collected retrospectively.

### Data analysis

For this observational study, descriptive statistics on baseline variables were reported as frequency (percentage) for categorical variables and as median, or range [minimum and maximum]) for continuous variables. Cumulative (e.g. all patients who achieve each stage) and conditional proportions (e.g. all patients who meet prior and current stage of the cascade) were calculated using the appropriate numerators and denominators from each stage, and tabulated with Microsoft Excel version 16.0.

## Results

A total of 603 patients were enrolled in the UP-PGH SAGIP treatment hub during the study period. Nineteen patients were excluded—18 were not officially enrolled as part of the treatment hub and 1 had a negative confirmatory Western blot. Thus, 584 patients were included in the study cohort. The study population was predominantly male (532/584, 91.1%), with a median age of 29 years (range, 0.17–68 years). Sexual contact, particularly male-to-male sex, was the most common mode of transmission (325/584, 55.6%). Among the 52 HIV-diagnosed female patients, 17 were pregnant at the time of first consult. At first consult, 58.2% also had advanced HIV disease, and 19.4% fulfilled criteria for late HIV diagnoses. The initial median CD4 count was 92.5 cells/uL (1–1483 cells/uL). Only 132 (22.6%) patients had a viral load determination prior to initiation of ART. Other characteristics of the cohort are summarized in Table 1.

**Table 1:** Characteristics of study participants.

Baseline Characteristic	N (% or median)
Sex, n (%)	584 (100)
Male	532 (91.1)
Female	52 (8.9)
Age in years, n (%)	
<15	8 (1.4)
15–24	120 (20.6)
25–34	320 (54.8)
35–49	121 (20.7)
≥50	15 (2.6)
Pregnant, n (% of all females)	17 (32.7)
Modes of HIV transmission, n (%)	
Sexual Contact	
Male-Female sex	77 (13.2)
Male-Male sex	325 (55.6)
Sex with males and females	122 (20.9)
Blood/blood products	0
Sharing of needles	1 (0.2)
Needlestick injury	0
Mother to child	8 (1.4)
No data	51 (8.7)
**Description of HIV/AIDS**	
WHO Clinical Stage, n (%)	
1	170 (29.1)
2	45 (7.7)
3	122 (20.9)
4	229 (39.2)
Not reported	18 (3.1)
Initial CD4 count median, cells/uL (range)	92.5 (1–1483)
With baseline HIV viral load prior to starting ART, n (%)	132 (22.6)

WHO–World Health Organization

Of the 584 patients included in the study, 581 (99.5%) were linked to care and 555 (95.0%) were started on ART (Table 2). Three patients who were enrolled did not arrive on their scheduled initial consult. The time to linkage to care of 84 patients were undetermined as they were transferred from other treatment hubs. Majority of patients had a CD4 count determination done within three months after diagnosis (Table 2). Four hundred sixty of the 584 (78.8%) patients were retained in care at the time of study review. Of the 460 patients, 400 (87.0%) were maintained on a fixed dose combination of lamivudine (3TC), tenofovir (TDF), and efavirenz (EFV), and 59 (12.8%) were given other combination ART. One patient was not maintained on any regimen at the time of study pending evaluation for possible cytomegalovirus (CMV) retinitis after stopping ART and being lost to follow-up.

**Table 2:** Outcomes at each stage of HIV care.

Stage of HIV Care	Total N, (%)
Enrolled in care, N (%)	584 (100)
Linkage to care (N = 581), n (%)	
<3 months	386 (66.4)
3–6 months	35 (6.0)
6–9 months	9 (1.6)
9–12 months	12 (2.1)
>12 months	55 (9.5)
Cannot be determined	84 (14.4)
ART initiation (N = 581), n (%)	
<3 months	388 (66.8)
3–6 months	54 (9.2)
6–9 months	22 (3.8)
9–12 months	16 (2.8)
>12 months	65 (11.2)
Cannot be determined	10 (1.7)
No ART initiation	26 (4.4)
Retention in care (N = 581), n (%)	
Died	15 (2.56)
Lost to follow-up	62 (10.6)
Transferred	44 (7.57)
Retained in Care	460 (79.2)
Alive and on ART	459 (79.0)
ART stopped	1 (0.2)
Viral suppression (N = 280), n (%)	
Yes	266 (95.0)
No	14 (5.0)

ART–Antiretroviral therapy

About half of patients (29/62, 46.8%) were lost to follow-up within three months of their initial consult. The remaining patients (33/62, 53.2%) were lost to care beyond the first three months with the greatest proportion (8/33, 24.2%) lost to follow up 15 months after initial consult.

Four hundred fifty-seven of the 460 patients who were retained in care were Philhealth members, but only 384 (84%) had up-to-date payment status at the time of review.

**Fig 1:**
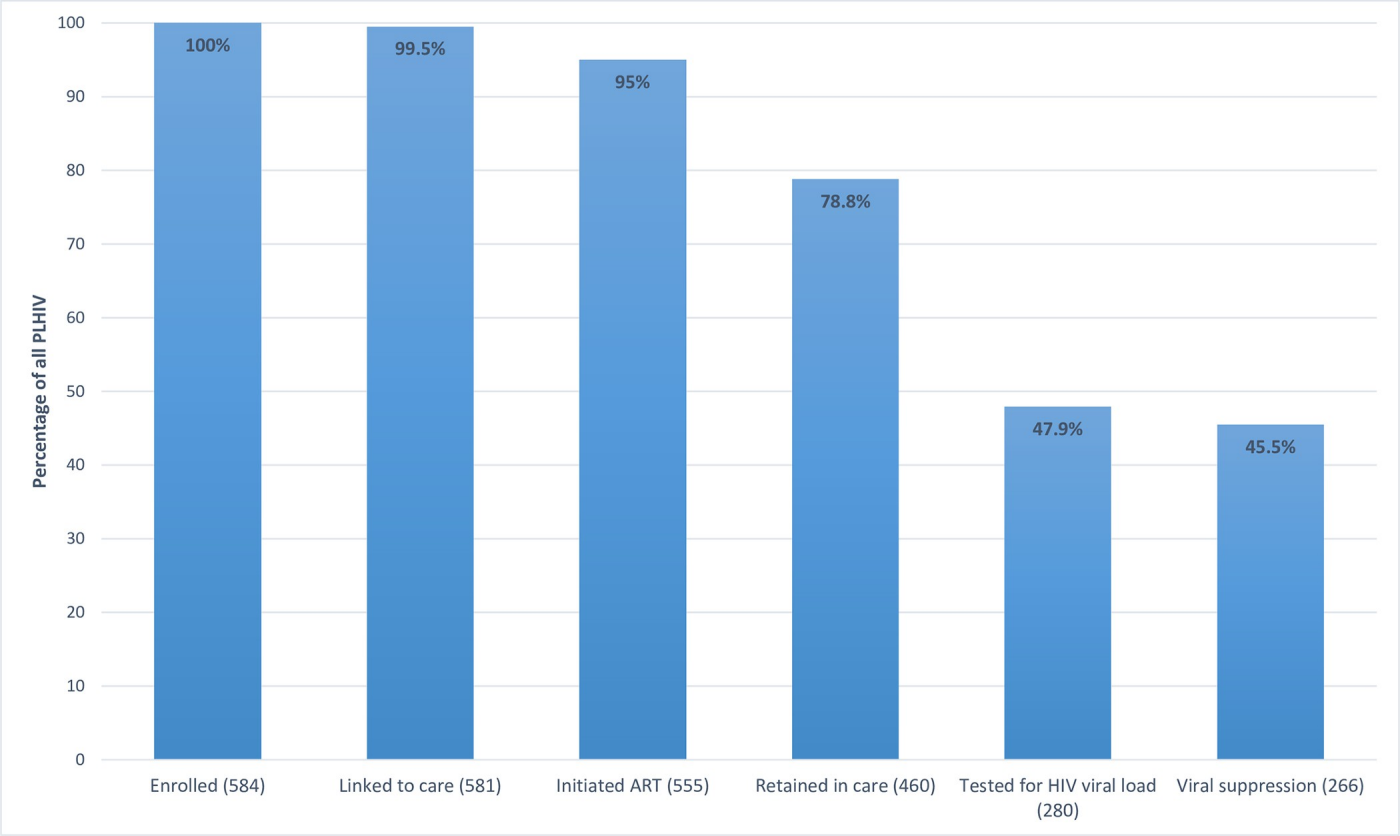
Cumulative retention at each stage of HIV care at the UP-PGH SAGIP Unit.

Using conditional proportions of the patients retained in care and on ART (n = 459), 280 (61.0%) had HIV viral load testing at least one year after ART initiation, with 266 (57.9%) patients achieving viral suppression (i.e. 45.5% of the original cohort) (Table 2; Fig 1). A subset of patients (n = 21) had HIV viral load determination done at <1 year after initiation, with majority of patients (20/21) achieving viral suppression. One patient achieved viral suppression after an undetermined amount of time from ART initiation. The remaining patients had either no viral load test result in their records, had pending results, or were still scheduled to undergo testing. Cumulative retention at each stage of HIV care from enrollment to viral suppression is shown in Fig 1.

## Discussion

In our study, we found that almost all patients enrolled at the UP-PGH SAGIP treatment hub were linked to care and initiated ART. However, there was a substantial decrease in the proportion of patients who were retained in care, had timely viral load determination, and attained virologic suppression. Only 45.5% of patients enrolled at the UP-PGH SAGIP treatment hub followed all stages of care from time of enrollment to viral suppression. Interestingly, the SEARCH Universal test and treat (UTT) trial conducted in Kenya and Uganda which aimed to reduce HIV incidence and improve community health showed that even in a rural, resource poor setting, the UNAIDS 90–90–90 targets are achievable reaching ‘92–95–90’ and 79% population-level viral suppression after 3 years. This was accomplished through community engagement, integration of HIV with other multi-disease services, rapid ART start upon HIV diagnosis, and patient-centered, streamlined care [20]. In the Philippines, Republic Act No. 11166, a law approved in 2019 to advocate for PLHIV, mandates the adoption of a similar multi-sectoral approach in response to the ongoing HIV epidemic; however, its implementation and application on the ground appears insufficient at this time. In addition, there are other gaps that need to be addressed including (1) outdated HIV and AIDS legal framework for prevention and control; (2) poor HIV and AIDS information, education and communication program; (3) existing barriers to condom access and low condom use; and (4) declining external funding support yet increasing new HIV cases [21, 22].

In our cohort, timely HIV testing remained a challenge as majority (77.6%) of patients had either late or advanced disease. Our figure is much higher than data from the Philippine DOH Epidemiology Bureau [18] where only 58% of newly diagnosed cases were classified in these categories, and from published data in the South East Asian region [14] where these cases comprised only 40.2%. The median baseline CD4 count in our study was 92.5 cells/uL, lower than the national median baseline of 133 and 132 cells/uL, in 2015 and 2016, respectively [18]. That many of the patients enrolled in SAGIP have late stage HIV disease is not surprising, and may be reflective of 1) the difficulty in accessing healthcare and/or 2) the reluctance of the young MSM population to get tested.

UP-PGH is a tertiary referral hospital that caters to the marginalized and underserved populations, who often seek consult only when they are critically ill [e.g. with an opportunistic infection (OI)] or symptomatic, during the late stages of their illness. However, a recently published study [13] among young cis-MSM in a community-based treatment hub, also showed that a good proportion (35%) of newly diagnosed cases in their cohort presented with advanced HIV disease. This suggests that similar factors in both community- and hospital-based hubs played a role in the delay in diagnosis. The authors surmised that these factors are directly related to the high proportion of young MSM PLHIV present in both settings. Other studies [13, 23, 24] have identified the young MSM population as an “at risk” population because they encounter many barriers unique to them that delay HIV testing. For example, in one study [24] fear of provider discrimination was a barrier to accessing HIV treatment services, and resulted in participants hiding their sexual orientation and/or gender identity. Confidentiality concerns also included clinic physical arrangements that segregated HIV testing from other health services, fear that healthcare providers would publicly disclose their status, and concerns that peers would discover they were getting tested [24]. As such, solutions to address these barriers specific among the MSM population need to be tackled by future studies.

### Linkage to care

Linkage to care of patients within three months of diagnosis was maintained at close to 80% from 2015 to 2017. It is concerning that 10% of patients were linked to care more than 12 months after HIV diagnosis. At the UP-PGH SAGIP treatment hub, patients who screen positive using an HIV point-of-care test are referred to a SAGIP provider on the same day. Patients referred by other healthcare personnel or testing centers with either a positive rapid HIV test or confirmatory test are also referred to a SAGIP provider on the day of their first visit. Factors that contribute to delayed linkage to care are likely multifactorial and may include late referrals (i.e. healthcare personnel opt to wait for the confirmatory test results, causing a delay of 1 to 3 months), hesitance in pursuing HIV care due to stigma, and limited resources of referred patients (i.e. patients who need to travel from the provinces often need transportation and accommodation expenses). Hesitance in pursuing care may also stem from factors deeply ingrained in Filipino culture that inadvertently perpetuate stigma, such as religious and fatalistic beliefs, a culture of collectivism, and machismo attitudes [13, 25].

### ART initiation

95% (555/584) among those enrolled started ART. This outcome is much higher compared to national data (76%) [8], and compared to a community-based hub where only 78% (2460/3137) initiated ART [13]. Our cohort also had a better rate of ART initiation compared to a similar population in Indonesia, of whom only 86.2% started ART [15].

Only two thirds (66.8%) of patients initiated ART within three months of their HIV diagnoses. Although SAGIP providers strive to start early ART for all patients if there are no contraindications, factors leading to delayed ART initiation is likely complex and may include patient’s preference, as well as a delay in laboratory work-up and specialty referrals. The SAGIP hub has no laboratory capacity. As such, facilitating diagnostic tests depend upon the patient’s own initiative, financial capability, and even turn-around time of the laboratory they choose to go to. Accommodation by subspecialty services (e.g., ophthalmology, dermatology) at the UP-PGH are also often delayed due to congestion of these outpatient services. Only a few patients have the capacity to go to private clinics where they pay out of pocket. The prolonged turn-around-time of HIV confirmatory testing likely also contributes to the delay in ART initiation, as patients often prefer to wait for confirmation despite the presence of an OI or AIDS defining illness. Another possible barrier is the simultaneous diagnosis and treatment of an active OI which, based on current guidelines, often preempts immediate initiation of ART to avoid complications (i.e. immune reconstitution inflammatory syndrome). Improvement of services including the provision of more accessible or expedited laboratory testing, and conversion of the hub to a multidisciplinary clinic where patients can be evaluated by subspecialty services more promptly to achieve a one-stop shop strategy [13], may lessen the delay in ART initiation. The importance of early ART initiation to achieve viral suppression, decrease HIV transmission and reduce new infections, should also be repeatedly discussed and emphasized to patients who are hesitant to initiate ART [26]. These factors need to be studied further as delay in ART is associated with increased likelihood of progression to AIDS and greater risk of ongoing HIV transmission [27].

### Retention in care

The UP-PGH SAGIP hub had a smaller proportion of patients retained in care and maintained on ART after a minimum of one year follow up compared to national data in 2016 (79% vs. 92%) [18]. A Kaplan-Meier survival analysis (S1 Fig) evaluating time to event (e.g. lost to care) showed that by 18 months, around 10% (e.g. 59/581) were lost to follow up and by the end of 48 months, only around 76% were retained in care; future studies should determine the specific reasons for poor follow- up. Although we were unable to evaluate the factors associated with attrition in this study, other reports have identified the MSM population as a vulnerable group having increased odds of poor retention in care because of fears driven by layered stigma and discrimination, lack of social and financial support, and lack of employment opportunities [23]. A prospective cohort study done in Indonesia showed comparable results to our study with only 75.4% of participants who initiated ART being retained in care [15]. Other possible reasons for poor retention in care may include lack of resources to sustain follow-up in a tertiary hospital, distance of the treatment hub from the patients’ residence, and absence of a systematic tracking system. In addition, although 87% of those on an EFV-based regimen were retained in care, choice of ART, especially if associated with adverse effects, may impact adherence and retention in care. Efavirenz is traditionally associated with sleep disturbances, headache, and rashes; these may have also contributed to the poor retention in care for the remainder (n = 124).

### Viral load testing and virologic suppression

61% of patients retained in care and on ART (47.9% of all enrolled patients) had their HIV viral load done at least one year after ART initiation. This figure is higher compared to the reported national data in 2020 which showed that only 17% of PLHIV on ART were tested for HIV viral load. The rate of virologic suppression in our cohort (95%) was comparable to both national [8] (94%) and community-based data [13] (84%). Our data regarding subsequent viral load testing is similar to a local study in an urban hospital [28] where only a small proportion of patients [31 of 77 (40.2%)] had a subsequent test for viral load. In that study, the proportion of patients who were virologically suppressed was also lower compared to our cohort [24/31 (77.4%) vs. 266/280 (95%)]. In another study in Indonesia, of 71.1% patients retained in care who had a viral load test done in six months, 90.5% achieved viral suppression [15], which is more comparable to our study. This result is in line with the overall goal of viral suppression by six months to a year, and the UNAIDS target of 90% of all those on ART being virally suppressed by the year 2020 [4].

There is, however, much room to improve with regards to this stage of the HIV cascade. Half of our cohort (52.1%) were not tested for virologic suppression. All HIV providers order this routinely at 6 months per national guidelines, but many PLHIV are unable to comply. The cost of the test ranges from Php 4,900.00 to Php13,900.00 depending on the laboratory. This amount may be too heavy for patients to shoulder, or deemed more appropriate to use for more urgent needs such as hospitalization, medications for co-morbidities, or other expenses. Beginning in 2010, the Philippine Health Insurance Corporation (PhilHealth), a government funded initiative provided an Outpatient HIV/AIDS Treatment (OHAT) package with an annual reimbursement of Php 30,000.00 per patient, released in four quarterly payments of Php 7,500.00, which aims to cover costs of medicines, professional fees, as well as laboratory exams including HIV viral load. Despite this available resource, PLHIV still encounter barriers including lengthy administrative processes and unclear procedures [14], explaining the low number of patients with HIV viral load results in our study. Patients with OHAT packages must also wait at least one year after initiation of ART for their viral load test to be free of charge. Improvements in access to and delivery of the OHAT package must be made in order to facilitate its use among PLHIV.

### Limitations

Our study has several limitations. First, UP-PGH SAGIP is a tertiary care treatment hub, and the findings may not be generalizable to other smaller HIV clinics in the country. We were also unable to compare our cascade with a similar hospital-based tertiary center. Second, around 70% of PLHIV enrolled are from external referrals, and we were unable to accurately measure the first stage in the HIV care cascade (e.g. the proportion of people who are tested and found to be positive for HIV). Third, the proportion of those linked to care may not represent an accurate proportion as these patients were recruited at a care clinic. The lack of a system to track patient movements may have inaccurately classified some patients as lost to follow-up when they might have transferred to another treatment hub or died. Finally, we were unable to assess risk factors that led to good retention in care, or conversely, attrition from care. Nevertheless, this is the only study that specifically looked at the HIV care cascade after implementation of the test-and-treat strategy in an HIV treatment hub of a tertiary hospital with a substantial sample size.

## Conclusions

More than 90% of patients enrolled at the UP-PGH SAGIP treatment hub are linked to care and initiate ART, exceeding the set goal. However, there is a substantial decrease in the number of patients who are subsequently retained in care, tested for HIV viral load, and achieve viral suppression. We surmise there are many potential barriers that lead to delayed testing (e.g. financial constraints, limitations of the OHAT, lack of laboratory capacity in the SAGIP hub), ART initiation (e.g. prolonged time to HIV confirmation, presence of untreated OI’s), and poor retention in care (e.g. lack of access to the clinic, stigma) which need to be identified by future studies. In the Philippine context, in order to achieve the goals of the cascade of care, support from the different sectors is essential. Maximizing use of platforms available to key HIV populations, such as internet-based interventions, would greatly complement existing government programs, since youth are extremely active in social media and online dating sites and applications. Similarly, given the demographic of PLHIV seeking services in SAGIP hub, creation of a community-based clinic, outreach programs, and/or exploration of other services such as use of short message service (SMS) or telemedicine may improve health service delivery and the cascade of HIV care. In conclusion, implementation of a community-based, patient-centered approach similar to the SEARCH-UTT but tailored to our own circumstances in order to reach the goals of the HIV cascade, with focus on the key sub-group of young, MSM, PLHIV, may be beneficial.

## Supporting information

S1 Fig Kaplan-Meier survival analysis. (TIF)
