# Supplementary figures and images for: A descriptive retrospective study on HIV care cascade in a tertiary hospital in the Philippines
Source: PLoS One. 2023 Jan 31;18(1):e0281104. doi: 10.1371/journal.pone.0281104 (PMC9888700; doi:10.1371/journal.pone.0281104)

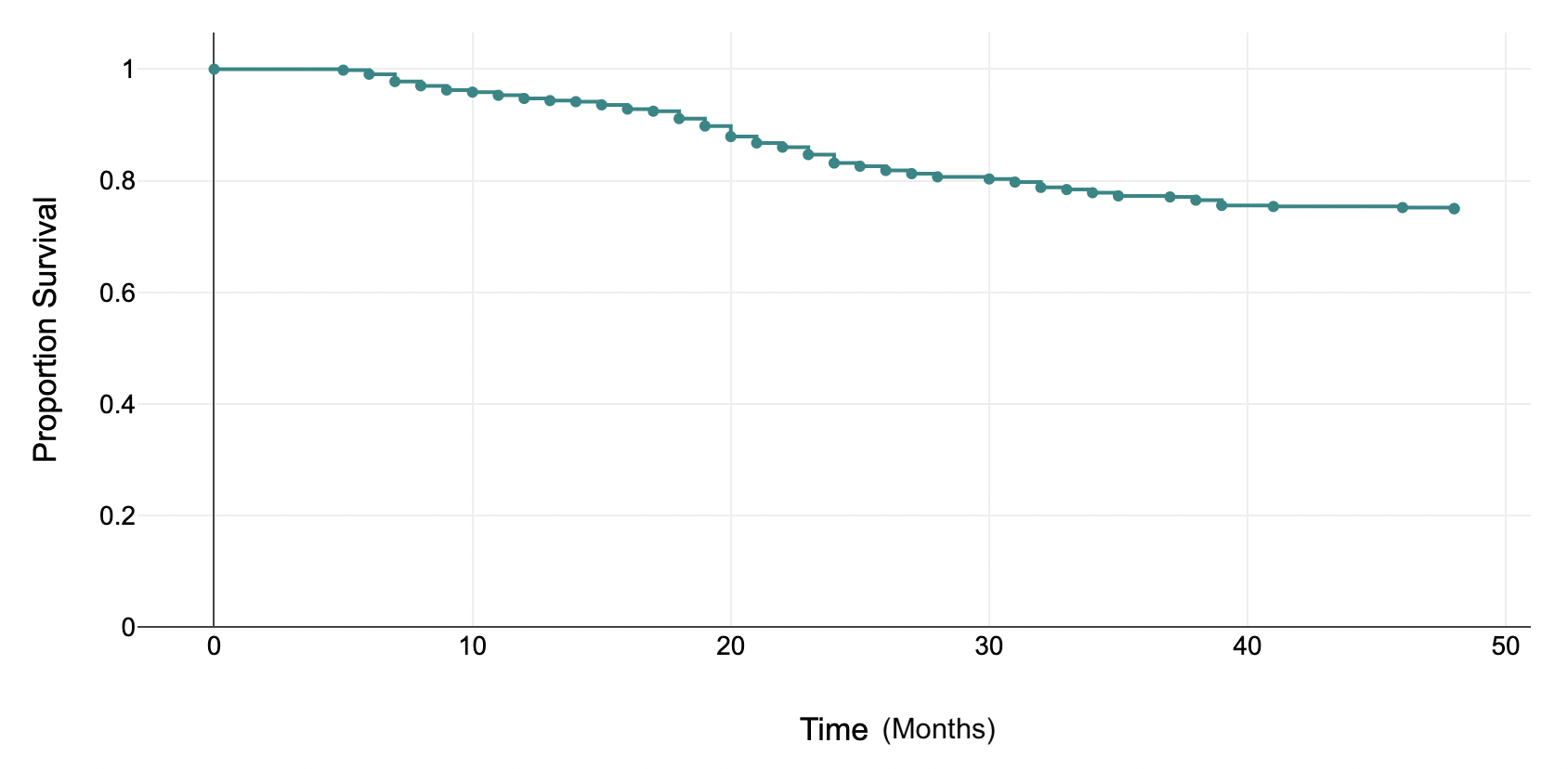

Supplement: S1 Fig — (TIF) [file pone.0281104.s001.tif]
